# Highland Barley and Its By-Products Enriched with Phenolic Compounds for Inhibition of Pyrraline Formation by Scavenging α-Dicarbonyl Compounds

**DOI:** 10.3390/foods10051109

**Published:** 2021-05-17

**Authors:** Dianwei Zhang, Pei Zhu, Luxuan Han, Xiaomo Chen, Huilin Liu, Baoguo Sun

**Affiliations:** Beijing Advanced Innovation Center for Food Nutrition and Human Health, Beijing Engineering and Technology Research Center of Food Additives, Beijing Technology and Business University, Beijing 100048, China; zhangdianwei@btbu.edu.cn (D.Z.); zp603541560@163.com (P.Z.); hlx19951112@163.com (L.H.); chenxiaomo05@163.com (X.C.); sunbg@btbu.edu.cn (B.S.)

**Keywords:** advanced glycation end products, 3-deoxyglucosone, glyoxal, methylglyoxal

## Abstract

Pyrraline, a typical kind of advanced glycation end product, has been found to contribute to the development of pathologies associated with ageing and diabetes mellitus. In the study, phenolic compounds extracted from highland barley whole grain (HBWG) and vinasse (HBVN) were used to inhibit pyrraline formation in a simulated food. The optimal extraction condition for HBWG and HBVN was using 8 mL of 50% acetone solution at 50 °C for 60 min. The extraction and identification of phenolic compounds from HBWG and HBVN were performed by UPLC–PAD–MS/MS. The inhibitory effects of pyrraline in the simulated food were 52.03% and 49.22% by HBVN and HBWG, respectively. The diphenyl picrylhydrazyl radical- and ferric-reducing ability of plasma assays was used to evaluate the antioxidant activity of the extracts. The main inhibition pathways and molecular mechanism of phenolic compounds on pyrraline regulation were explored by scavenging α-dicarbonyl compounds. The study demonstrated that highland barley and its by-products can potentially be used as a functional food to regulate pyrraline formation during food processing.

## 1. Introduction

The Maillard reaction, also known as nonenzymatic browning or protein glycation, was originally discovered by Louis Maillard [1]. A series of chemical reactions occur between sugars and proteins, resulting in a yellowish-brown color change during food heating, processing, and storage [2]. The Maillard reaction leads to a lot of positively food-related colors, flavors, and aromas, which also occur in vivo. It is seen in conjunction with accelerated aging and diabetic complications [3,4]. However, controlling the negative effects of the Maillard reaction on food quality and safety is an urgent problem. Because of the potential health risks associated with advanced glycation end products (AGEs), people are increasingly concerned about the level of foodborne AGEs.

Many studies have shown that the Maillard reaction is initiated with the attachment of the aldehyde function of acyclic glucose to a protein amino group using nucleophilic addition to form an aldimine of a Schiff base [5]. The stable Amadori product is formed by Schiff base rearrangement [6], which could undergo multiple chemical rearrangements to form high reactivity of carbonyl compounds. These compounds react again with free amino groups of proteins, resulting in the production of advanced glycation end products (AGEs) [7]. Several AGEs have been proposed, such as N^ε^-(carboxymethyl)-L-lysine [8], N^ε^-carboxymethyl arginine [9], perlysines [10], crosslines [11], imidazolone [7], and vesperlysines [12]. Pyrraline, ε-2-(formyl-5-hydroxymethyl-pyrrol-1-yl)-L-norleucine, is one of the major compounds of AGEs formed in the final stage of the Maillard reaction [8]. It is generally accepted that the main compounds of AGEs are pathophysiologically derived from the ingestion of dietary N^ε^-carboxymethyllysine, but pyrraline studies are lacking.

α-Dicarbonyl compounds are the typical degradation products of reducing sugars and Amadori products for AGE formation during food processing, such as 3-deoxyglucosone (3-DG), glyoxal (GO), and methylglyoxal (MGO) [13]. These compounds induce inter- and intra-crosslinks in proteins, leading to their structural and functional changes. Pyrraline may be formed from α-dicarbonyl compounds and the ε-amino group of amino acid or proteins. Portero-Otin et al. [14] reported higher levels of urinary pyrraline in patients with diabetes. We quantified pyrraline in milk and milk powder using core–shell nanoparticles as absorbents coupled with high-performance liquid chromatography, which was constructed of metal–organic frameworks as core and molecularly imprinted polymers as shell [15]. Convenient and fast optical-based sensing technology was used for the sensitive detection of pyrraline in our lab [16]. However, there was a lack of research on the inhibition of pyrraline during food processing. Therefore, we focused on the inhibition of foodborne pyrraline by α-dicarbonyl compounds.

Highland barley, the largest coarse cereal in northwest China, has attracted much attention for its unique nutritional value as a bioactive phytochemical [17]. Recently, whole grain intake has been found to reduce the risk of chronic diseases possibly due to its rich bioactive phytochemicals. Many studies have shown that bioactive phytochemicals could combat oxidative stress in the body by maintaining a balance between oxidants and antioxidants [18,19]. Emerging academic research has confirmed that highland barley whole grain (HBWG) contains an abundance of native phytochemicals. In particular, an average phenolic content of 333.9−460.8 mg is the most abundant in HBWG [20]. The addition and synergism of these phytochemicals endow HBWG with potential functions, such as antihyperglycemic, antihyperlipidemic, and anticancer activities [21,22]. Highland barley vinasse (HBVN) is the residue of highland barley grains after fermentation and wine making, which is usually discarded as waste or used to produce animal feed. The potential applications of HBWG and HBVN in the inhibition or degradation of pyrraline-induced chronic disease have rarely been studied.

In the study, phenolic compounds extracted from HBWG and HBVN were used to inhibit foodborne pyrraline associated with the Maillard reaction in food processing. The extraction and identification of phenolic compounds from HBWG and HBVN were studied by an ultraperformance liquid chromatography–photodiode array detector coupled with mass spectrometry/mass spectrometry (UPLC–PAD–MS/MS). The inhibition effect of HBWG and HBVN on pyrraline was performed by a simulation system based on glucose and L-lysine. The diphenyl picrylhydrazyl radical- and ferric-reducing ability of plasma assays was used to evaluate the antioxidant activity of HBWG and HBVN. The main pathway and the molecular mechanisms of phenolic compounds on pyrraline inhibition were explored. Several studies have shown that α-dicarbonyl compounds are major intermediates in the formation of AGEs by protein glycation [23,24], leading to associated α-oxoaldehyde-derived AGE formation. Therefore, the pathway of α-dicarbonyl compounds is an important topic of discussion in the study.

## 2. Materials and Methods

### 2.1. Chemicals and Materials

Glucose, disodium hydrogen phosphate, sodium dihydrogen phosphate, methanol, and ammonia (all of analytical grade) were purchased from Xilong Scientific Co., Ltd. (Guangdong, China). L-lysine (analytical grade) was purchased from Sinopharm Chemical Reagent Co., Ltd. (Beijing, China). Trifluoroacetic acid and acetonitrile (all of chromatographic grade) were purchased from J&K Scientific Ltd. (Beijing, China). Pyrraline (standard) was purchased from Beijing Pinellia Technology Development Co., Ltd. (Beijing, China). The analytical-grade standards of α-dicarbonyl compounds of 3-DG, GO, and MGO were purchased from Sigma-Aldrich (Zwijndrecht, The Netherlands).

### 2.2. Equipment

Samples were weighed by an ME104 electronic balance (Mettler Toledo Instrument Co., Ltd., Shanghai, China). Samples were mixed with an MXF Vortex mixer (Scilogex, Rocky Hill, CT, USA). Samples were heated and extracted by a DF-101S Hermostatic heating magnetic stirrer (Gingili City Yuhua Instrument Co., LTD, Gongyi, China). Samples were dried using an R-210 rotary evaporator (Büchi Labortechnik AG, Flawil, Switzerland). Samples were cleaned by an Oasis^TM^ HLB solid-phase extraction (SPE) column (Waters, Milford, MA, USA. Pyrraline and 3-DG were determined by HPLC with a diode array detector (DAD, Shimadzu, Kyoto, Japan). The main phenolic compounds were separated using an ultraperformance liquid chromatography–photodiode array detector coupled with mass spectrometry/mass spectrometry (UPLC–PAD–MS/MS, Thermo Scientific, Waltham, MA, USA).

### 2.3. Purification of Pyrraline by Solid-Phase Extraction

All samples were cooled prior to analysis. HLB SPE columns were preactivated by 3 mL of methanol and 3 mL of water. The cooled samples were transferred to an SPE column. The impurities were washed with 3 mL of water and 3 mL of methanol in turn. Then, pyrraline was eluted with 5 mL of methanol containing 5% ammonia, collected, and dried with nitrogen. Finally, the solution was dissolved in 2 mL of ultrapure water.

### 2.4. Determination of Pyrraline by HPLC

The determination of pyrraline was performed by an HPLC–DAD system (Shimadzu) based on the methods described by Portero-Otin et al. [14] with some modifications. The analysis was accomplished with an Inertsil ODS-SP column (4.6 × 250 mm, 5 μm). The mobile phase used 0.1% trifluoroacetic acid (TFA, *v*/*v*) in ultrapure water as solvent A and 50:50 (*v*:*v*) acetonitrile (ACN)/ultrapure water as solvent B. The gradient steps were 1–10 min, 0% B; 10–30 min, 0–15% B; 30–35 min, 15–20% B; 35–40 min, 20–100% B; 40–45 min, 0% B. The flow rate was 1 mL/min, and the injection volume was 5 μL. Detection was performed at a wavelength of 298 nm with a final time of 100 min.

### 2.5. Samples

The samples of HBWG and HBVN were from the local factory. The extraction of the whole grain of highland barley was based on the methods described by Meneses et al. [25] with some modifications. HBWG and HBVN weighed with 1 g samples each were extracted with 8 mL of 50% acetone solution at 50 °C for 60 min. The extract was centrifuged at 5000 rpm for 10 min, the supernatant was collected, and the insoluble component was extracted twice using the same method. The supernatant was merged and dried by a rotary evaporator. After nitrogen blowing, it was dissolved in 1 mL of ethanol and filtered through a 0.2 μm microfiltration membrane for UPLC–PAD–MS/MS analysis.

### 2.6. Phenolic Profile Using UPLC–PAD–MS/MS Analysis

The high-throughput UPLC–PAD–MS/MS method was used for the determination of 60 kinds of compounds in HBVN. The phenolic profile was determined using UPLC–PAD–MS/MS (Thermo Scientific). The phenolic compounds were separated using a Hypersil GOLD column (Thermo Scientific, 100 × 2.1 mm, 3 μm). Mobile phases A and B were 0.1% formic acid–ultrapure water and 0.1% formic acid–acetonitrile, respectively. The gradient was programmed as follows: 0 min, 95% B; 0–4 min, 95–90% B; 4–16 min, 90–10% B; 16–22 min, 10% B; 22–22.1 min, 10–95% B; 22.1–30 min, 95% B. The flow rate was 0.25 mL/min, the sample injection volume was 20 μL, and the PAD detector was set at 190–800 nm. The detection was carried out by MS in electrospray ionization (ESI) positive mode and scanned from *m*/*z* 0–1000 with the following parameters: capillary voltage, 4.0 kV; gas temperature, 300 °C; and nebulizer, 15 psi.

### 2.7. Determination of Phenolic Compound Scavenging by α-Dicarbonyl Compounds

The standards of phenolic compounds (5 mM) were mixed with 5 mM GO, MGO, and 3-DG for 36 min at 101 °C, respectively. The study used HPLC with an Inertsil ODS-SP column (4.6 × 250 mm, 4.6 μm) at a column temperature of 25 °C. The mobile phases A and B were 0.15% acetic acid and 100% acetonitrile, respectively. The gradient was programmed as follows: 0–10 min, 8–40% B; 10–12 min, 40–48% B; 12–13 min, 48–60% B; 13–15.5 min, 60–80% B; 15.5–20.5 min, 80% B; 20.5–25.5 min, 80–45% B; 25.5–30.5 min, 45–8% B; 30.5–35 min, 8% B. The flow rate was 0.8 mL/min, the injection content was 20 μL, and the wavelength was set at 313 nm.

### 2.8. Inhibition of Pyrraline Formation by Phenolic Compounds from HBWG and HBVN

An inhibition study was performed by adding HBWG and HBVN (1 mL) to the simulation system, respectively. We used the typical simulation system of L-lysine and glucose, and the preparation process for the model system was as follows [13]: 0.1 mol/L L-lysine and glucose were mixed with 0.1 mol/L phosphate-buffered solution (PBS, pH 7.4). The mixtures were incubated at 80 °C for 40 min and cooled immediately for further analysis. To evaluate the inhibition effect, the simulation systems were compared with a control experiment without the addition of HBWG.

### 2.9. Antioxidant Activities of HBWG and HBVN

The radical-scavenging activity was studied using the DPPH and FRAP methods. In the dark, 150 μL of HBWG or HBVN was mixed with DPPH (2.85 mL, 6.6 × 10^−5^ M) for 2 h, and then the absorbance was measured with UV detection at 515 nm. The inhibition effect of the DPPH method was calculated using Equation (1) [26]:

(1) $$Inhibition\;\left(\%\right)=1-100*\frac{As}{Ac}$$

where *As* and *Ac* are the absorbance values of the sample and blank, respectively. In the FRAP method, we used S0116 test kits for analysis of radical-scavenging activity. In the acidic conditions, ferric tripyridyl triazine TPTZ-Fe (III) was reduced to ferrous tripyridyl triazine TPTZ-Fe (II) by antioxidant activity. The concentration of TPTZ-Fe (II) was determined with UV detection at 593 nm.

### 2.10. Statistical Analysis

The results are expressed as the means ± standard deviation from three independent determinations. Analysis of variance was used to establish any significant differences (*p* < 0.05) between the applied treatments using the SPSS software package (version 17.0, SPSS Inc., Chicago, IL, USA).

## 3. Results and Discussion

### 3.1. Optimization of Different Extraction Conditions of Phenolic Compounds from HBWG and HBVN

**Figure 1:**
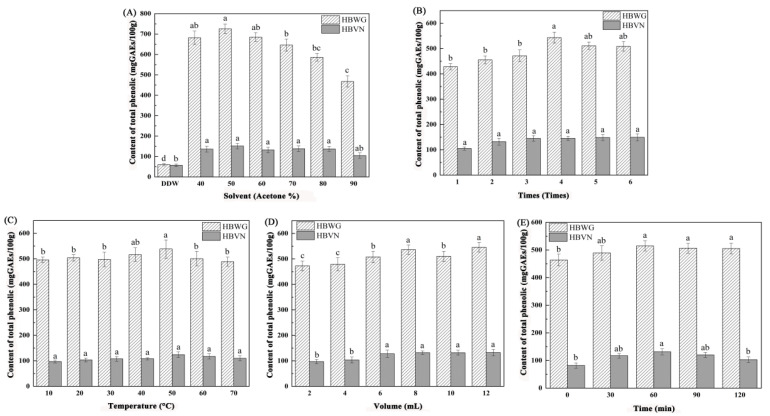
Extraction conditions of HBWG using the total phenolic compounds as the evaluation criterion. (**A**) Solvent, (**B**) Times, (**C**) Temperature, (**D**) Volume, and (**E**) Time. Error bars are standard deviations (*n* = 3). Different letters above the columns represent significant difference at *p* < 0.05.

The total content of phenolic compounds from HBWG and HBVN was evaluated by gallic acid with different extraction solvents. Preliminarily, our group studied that acetone was a good solvent for the extraction of phenolic compounds [27]. Therefore, we chose 40%, 50%, 60%, 70%, 80%, and 90% acetone for the extraction solvents and compared them with water (Figure 1A). When using 50% acetone as the extraction solvent, the extracted amount of phenolic compounds from HBWG was 725.97 mg/100 g, which was higher than that of the other solvents. When the acetone concentration was 50–70%, the extraction efficiency of phenolic compounds from different sources was relatively high, especially in the substrate containing protein, because acetone aqueous solution could effectively degrade the complex of polyphenol and protein. However, when the amount of acetone increased, other substances in HBWG might have been extracted, which hindered the extraction of polyphenols. When the acetone content was 50%, the extraction content of phenolic compounds in HBVN was 146.3 mg/100 g (Figure 1A). However, it had a negative effect at acetone concentrations greater than 50%. In the protein-based matrix, 50–95% acetone aqueous solution had relatively good extraction efficiency for different sources of phenolic compounds. Because acetone aqueous solution can effectively degrade the polyphenol–protein complex, when the amount of acetone increased, other impurities were extracted from the samples to inhibit the extraction of phenolic compounds. Therefore, we chose 50% acetone for the extraction solvent.

The extraction times were optimized by 1, 2, 3, 4, 5, and 6 (Figure 1B). When the extraction time was 4, the extraction amount of phenolic compounds in HBWG reached a maximum value of 544.33 mg/100 g. With increasing extraction times, the content of phenolic compounds from HBWG increased, but there was no significant difference with an extraction time of 4. It can be seen from Figure 1B that the extraction amount of phenolic compounds did not increase significantly with the continuous increase in extraction time. When the extraction times were 4, the extraction amount of phenolic compounds in HBVN reached a maximum value. When the extraction times increased, the extraction amount of phenolic compounds did not increase significantly. Therefore, we chose the optimal extraction time of 4.

Figure 1C shows that the content of phenolic compounds increased with increasing extraction temperature. When the extraction temperature was 50 °C, the extraction amount of phenolic compounds in HBWG reached a maximum value of 538.11 mg/100 g. As the temperature continued to rise, the extraction amount of phenolic compounds in HBWG decreased, which may be due to the degradation of phenolic substances caused by the temperature increase. Therefore, 50 °C was selected as the optimal extraction temperature of phenolic compounds in HBWG. When the extraction temperature was 50 °C, the extraction content of phenolic compounds in HBVN reached a maximum of 124.00 mg/100 g, and the content of phenolic compounds decreased with increasing temperature (Figure 1C). Therefore, 50 °C was selected as the optimal extraction temperature for HBVN extraction.

When the extraction solvent volume was 8 mL, the extraction content of phenolic compounds in HBWG and HBVN reached a maximum of 536.53 mg/100 g and 132.21 mg/100 g, respectively (Figure 1D). The extraction volume of phenolic compounds did not increase significantly with increasing solvent volume. Too much extraction solvent would lead to waste of raw materials and environmental pollution. Therefore, the optimal extraction volume was 8 mL.

As shown in Figure 1E, when the extraction time of HBWG and HBVN was 60 min, the extraction amounts of phenolic compounds reached a maximum value of 514.60 mg/100 g and 131.31 mg/100 g, respectively. The content of phenolic compounds in HBWG decreased after continuous heating, which may be due to the degradation of phenolic compounds caused by the long heating time.

### 3.2. Antioxidant Activity of HBWG and HBVN Extracts

**Figure 2:**
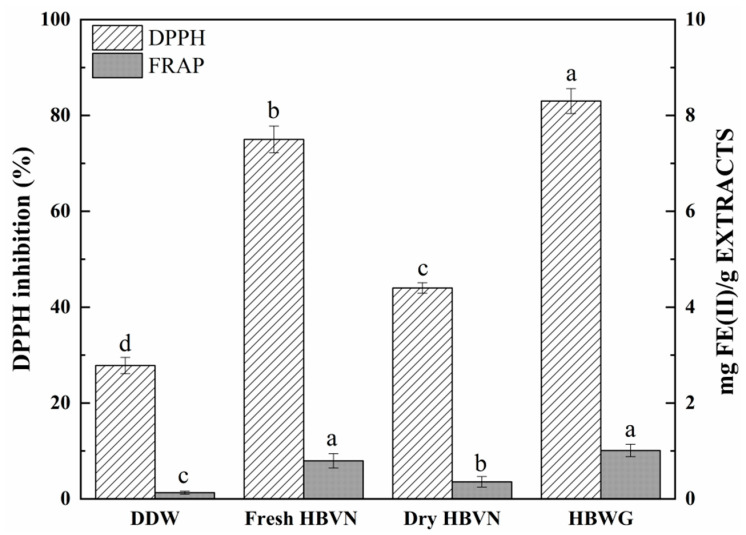
Evaluation of the antioxidant activity of HBWG and fresh and dry HBVN using the FRAP and DPPH methods. Error bars are standard deviations (*n* = 3). Different letters above the columns represent significant difference at *p* < 0.05.

Many studies have reported that AGE formation is viewed as a major contribution to oxidative damage in some tissues in chronic diseases, such as the pathogenesis of diabetic complications of eye lenses and nerves [28]. Therefore, oxidative stress is thought to be closely related to AGE formation, and antioxidant compounds are considered potential inhibitors of AGE formation [29]. The radical-scavenging activity uses fresh and dry HBVN to compare the antioxidant capacity. Fresh HBVN is wet and not conducive to preservation. After spray drying, HBVN is beneficial for long-term storage and utilization. Therefore, we used 0.05 g/mL of fresh and dry HBVN and 0.05 mg/mL of HBWG in the study. Figure 2 shows the scavenging activity of DPPH by using HBWG and fresh and dry HBVN extracts, at 83%, 75%, and 44%, respectively, which is higher than that using DDW at 27%. The antioxidant capacities of HBWG and fresh and dry HBVN extracts were 10.11, 7.94, and 3.57 mg FE(II)/g EXTRACTS for the FRAP method, respectively.

### 3.3. Inhibition of Pyrraline Formation by HBWG and HBVN Extracts in a Simulated Food System

**Figure 3:**
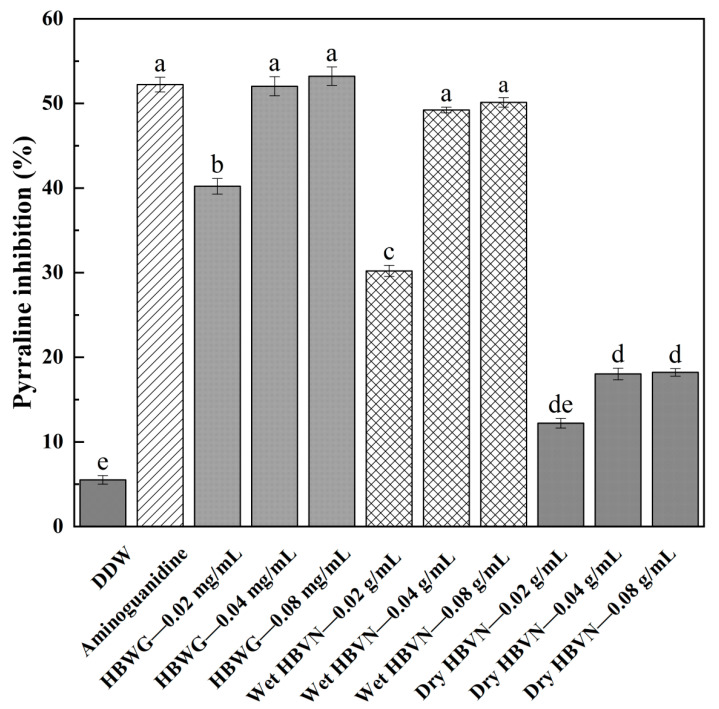
Inhibitory effect of HBWG and fresh and dry HBVN on pyrraline formation. Marker. Error bars are standard deviations (*n* = 3). Different letters above the columns represent significant difference at *p* < 0.05.

Figure 3 shows that the inhibitory effect of the HBWG extract was compared with that of aminoguanidine on pyrraline in the simulated food system. The inhibitory effects were studied at extract concentrations of 0.02, 0.04, and 0.08 mg/mL. When the HBWG addition was 0.04 mg/mL, pyrraline in the simulation system was reduced significantly with the best inhibition effect. The inhibition rate of pyrraline was 52.03% by HBWG extracts in the simulated system, which was not significantly different from that in the positive control aminoguanidine group. This result indicates that there are more kinds of phenolic compounds and that there is a higher content of phenolic compounds in HBWG.

The fresh and dry HBVN extracts were used to inhibit pyrraline formation in the model system. When the content of fresh HBVN was 0.04 g/mL, pyrraline in the model system was reduced significantly. The inhibition effect of fresh HBVN on pyrraline in the model system reached 49.22%, which was coincident with the positive control group of aminoguanidine. However, pyrraline inhibition was 11.77% by using dry HBVN with a concentration of 0.04 g/mL. This result indicates that there was a higher content of phenolic compounds in fresh HBVN than dry HBVN. However, phenolic compounds may be degraded by using high-temperature drying technology. This conclusion is consistent with the antioxidant activity of fresh and dry HBVN.

### 3.4. Isolation and Identification of the Main Functional Compounds from HBWG and HBVN Extracts by 50% Acetone

**Figure 4:**
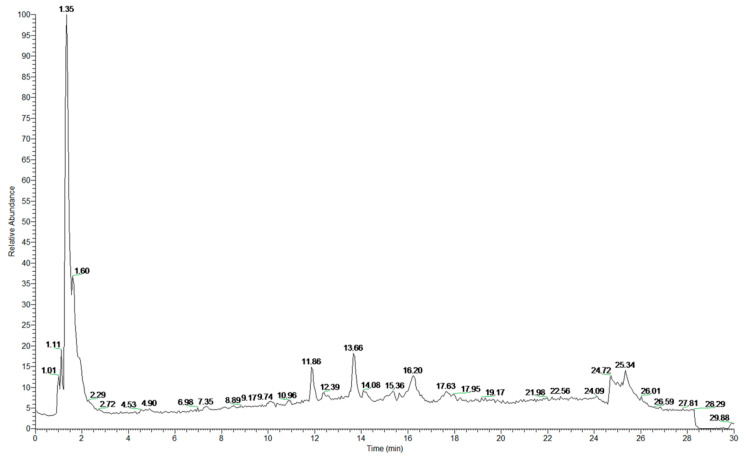
UPLC chromatographic separation of phenolic compounds from HBWG.

**Figure 5:**
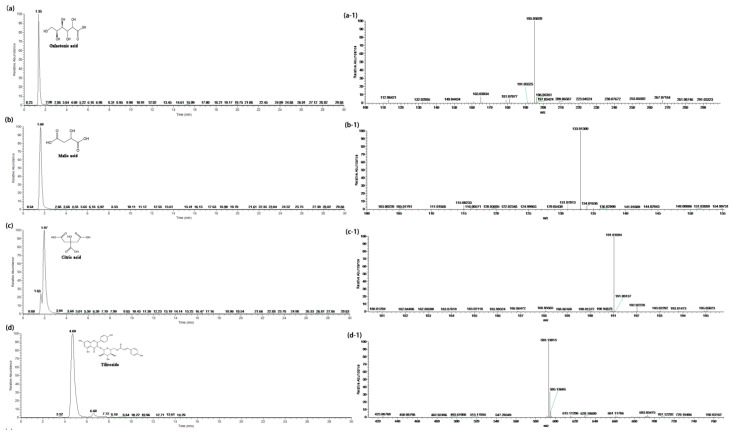
Identification of 14 kinds of compounds from HBWG by UHPLC–PAD–MS/MS. (**a**) UPLC chromatogram of galactonic acid, (**a-1**) MS for galactonic acid, (**b**) UPLC chromatogram of malic acid, (**b-1**) MS for malic acid, (**c**) UPLC chromatogram of citric acid, (**c-1**) MS for citric acid, (**d**) UPLC chromatogram of tiliroside, (**d-1**) MS for tiliroside.

Cereals contain many antioxidants, typically polyphenols, such as clove acid, vanillic acid, ferulic acid, caffeic acid, fenugreek acid, procyanidins, catechin, anthocyanin, quinine, flavones, flavanone, and aminophenol compounds. Previous studies have shown that highland barley contains many antioxidants, especially polyphenols and flavonoids [17]. Fourteen kinds of functional compounds from HBWG were identified by UPLC–PAD–MS/MS in the present study (Figure 4). Typically, functional compounds include galactonic acid, malic acid, citric acid, tiliroside, catechin, azelaic acid, apigenin, rutin, caffeic acid, ferulic acid, dodecanedioic acid, ibufenac, and dihome (Figure 5 and Figure S1).

**Figure 6:**
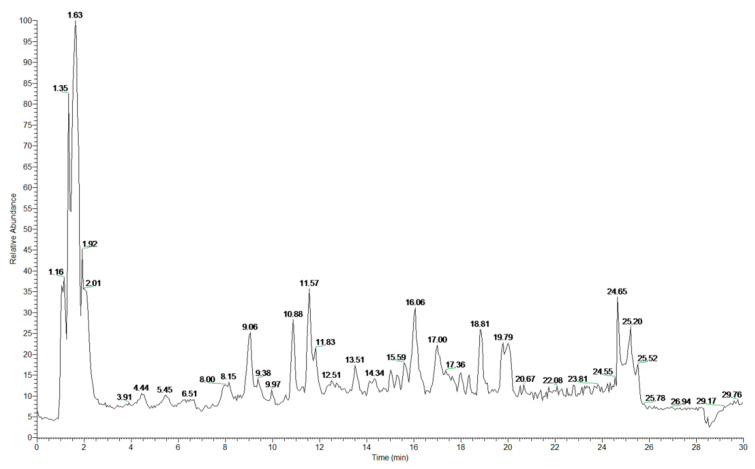
UPLC chromatographic separation of phenolic compounds from HBVN.

**Figure 7:**
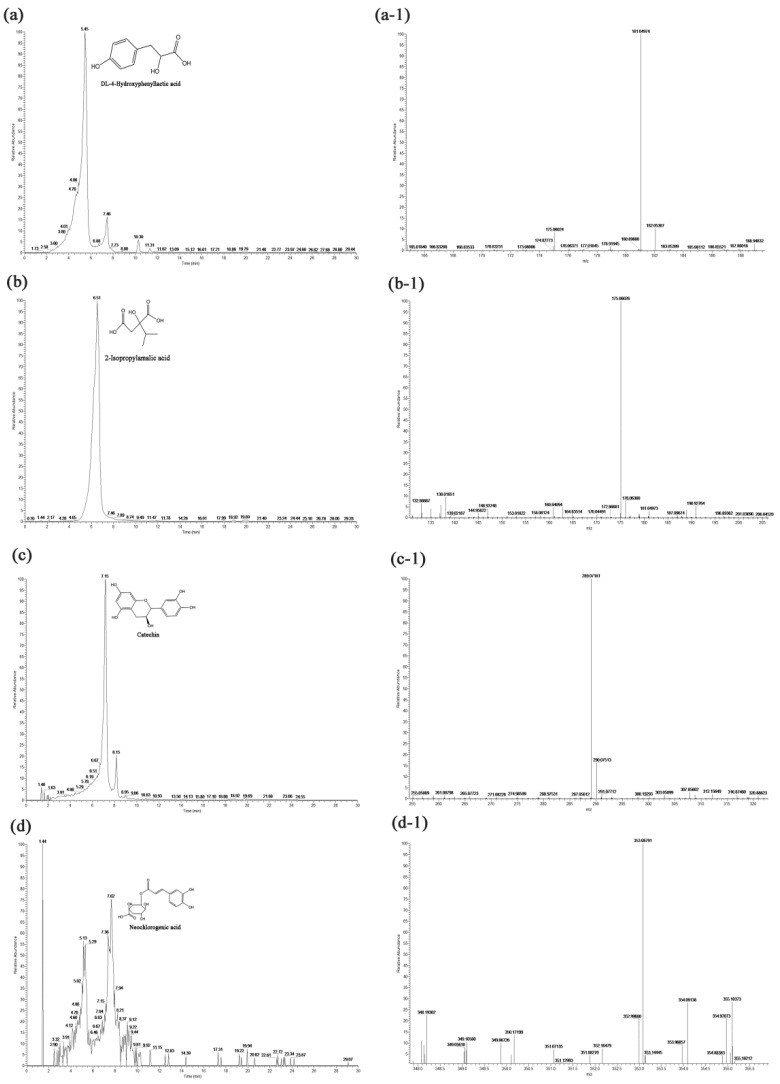
Identification of 21 kinds of compounds from HBVN by UHPLC–PAD–MS/MS. (**a**) UPLC chromatogram of DL-4-hydroxyphenyllactic acid, (**a-1**) MS for DL-4-hydroxyphenyllactic acid, (**b**) UPLC chromatogram of 2-isopropylmalic acid, (**b-1**) MS for 2-isopropylmalic acid, (**c**) UPLC chromatogram of catechin, (**c-1**) MS for catechin, (**d**) UPLC chromatogram of neochlorogenic acid, (**d-1**) MS for neochlorogenic acid.

According to the mass-to-charge ratio and the retention times of standards, 21 kinds of functional compounds from HBVN were identified by UPLC–PAD–MS/MS (Figure 6). Peaks 1–3 indicated DL-4-hydroxyphenyllactic acid, 2-isopropylmalic acid, and catechin, respectively. Peak 4 was neochlorogenic acid, 5 was caffeic acid, 6 was 2-hydroxycaproic acid, 7 was astilibin, 8 was rutin, 9 was 3-phenyllactic acid, 10 was isoquercetin, 11 was cynaroside, 12 was ferulic acid, 13 was neodiosmin, 14 was formononetin, 15 was azelaic acid, 16 was glycitein, 17 was luteolin, 18 was quercetin, 19 was apigenin, 20 was naringenin, and 21 was diosmetin (Figure 7 and Figure S2).

The proportions of functional compounds from HBWG and HBVN are shown in Table 1 and Table 2. The total contents of galactonic acid and malic acid from HBWG were higher than 60%, and the contents of DL-4-hydroxyphenyllactic acid, apigenin, 2-hydroxycaproic acid, quercetin, and 2-isopropylmalic acid from HBVN were higher than 10%, respectively. However, the contents of ferulic acid were only 0.43% and 1.58% from HBWG and HBVN, respectively. The main phenolic compound in highland barley is ferulic acid, which mainly exists in the binding type form. Therefore, the content of free ferulic acid was low. The content of acetone was more than 50%, so the extraction effect of phenolic compounds was variable, especially in the protein-based model system. There were more kinds of phenolic compounds detected in HBVN than in HBWG because more phenolic compounds were produced in grains after microbial fermentation.

### 3.5. Scavenging Experiment on α-Dicarbonyl Compounds by Phenolic Compounds from HBVN Extracts

During the nonenzymatic reaction system, the carbonyl group of the reducing sugar interacted with the ammonia group of the protein to form a Schiff base, which produced α-dicarbonyl compounds, such as GO, MGO, and 3-DG by Amadori rearrangement. These highly reactive α-dicarbonyl compounds reacted with ammonia groups of proteins to form AGEs. Therefore, we focused on phenolic compounds to scavenge and inhibit α-dicarbonyl compounds in the model system. Table 3 shows the main phenolic compounds that scavenge and inhibit α-dicarbonyl compounds at a concentration of 5 mM with 0.1, 0.2, and 0.5 mL. The resulting compound revealed a good inhibitory effect on pyrraline formation induced by α-dicarbonyl compounds. When using 0.5 mL of phenolic compounds, the scavenging effects on GO, MGO, and 3-DG were 43–54%, 42–57%, and 37–51%, respectively, indicating a good scavenging capacity of phenolic compounds for α-dicarbonyl compounds. When using a low concentration, the scavenging capacity was lower than when using a concentration of 0.5 mL (5 mM). The increase in concentration of phenolic compounds increased the scavenging capacity for α-dicarbonyl compounds. The major phenolic compounds had a good inhibitory effect of 40–55% on pyrraline formation with a concentration of 0.5 mL (5 mM). The inhibitory effects of phenolic compounds at three concentrations on food model systems in Table 3 show that the increase in concentration of phenolic compounds increased the inhibitory ability for pyrraline, which is consistent with the scavenging experiment.

## 4. Conclusions

In this study, the main phenolic compounds were extracted from HBWG and HBVN to assess the inhibitory effect on pyrraline formation. The optimal extraction condition for HBWG and HBVN was using 8 mL of 50% acetone solution at 50 °C for 60 min. The inhibitory effects of 52.03% and 49.22% exhibited good inhibitory effects by HBVN and HBWG. Separation and identification of the main phenolic compounds from HBWG and HBVN were performed by UPLC–PAD–MS/MS. Furthermore, the mechanism underlying the inhibition of HBVN extraction on the formation of pyrraline was discussed in detail. During simulated food processing, HBWG and HBVN extracts might inhibit the formation of foodborne pyrraline by two pathways: (1) scavenging of intermediate products of pyrraline formation, such as α-dicarbonyl compounds, and (2) inhibition of oxidative stress. This study not only was conducive to the recycling of resources but also provided a promising strategy for inhibiting the foodborne pyrraline formed in food processing with the addition of proper concentrations of HBWG and HBVN. Highland barley and its by-products enriched with phenolic compounds could potentially regulate pyrraline formation and show promise for future applications in the inhibition of other foodborne AGEs to effectively reduce glycation in foods and benefit those with chronic diseases.

## Figures and Tables

**Table 1:** Contents of functional compounds from HBWG were identified by UPLC–PAD–MS/MS.

No.	Compounds	Retention Time (min)	Content (%)
1	Galactonic acid	1.35	46.50 ^a^
2	Malic acid	1.60	16.56 ^b^
3	Citric acid	1.97	6.99 ^c^
4	Tiliroside	4.69	5.31 ^c^
5	Catechin	6.98	0.82 ^e^
6	Caffeic acid	7.35	2.93 ^cd^
7	Rutin	8.89	0.51 ^e^
8	Ferulic acid	9.17	0.43 ^e^
9	Azelaic acid	9.74	0.59 ^e^
10	Apigenin	10.96	1.99 ^d^
11	Octadecenoic acid	11.86	5.44 ^c^
12	Dodecanedioic acid	12.39	1.90 ^d^
13	Ibufenac	13.66	6.45 ^c^
14	Dihome	14.08	3.58 ^cd^

Data are expressed as means ± SD (*n* = 3). Different letters represent significant difference at *p* < 0.05.

**Table 2:** Contents of functional compounds from HBVN were identified by UPLC–PAD–MS/MS.

No.	Compounds	Retention Time (min)	Content (%)
1	DL-4-Hydroxyphenyllactic acid	5.45	15.59 ^a^
2	2-Isopropylmalic acid	6.51	8.12 ^b^
3	Catechin	7.15	1.97 ^c^
4	Neochlorogenic acid	7.62	0.08 ^d^
5	Caffeic acid	7.89	1.90 ^c^
6	2-Hydroxycaproic acid	8.15	14.18 ^a^
7	Astilibin	8.42	0.14 ^d^
8	Rutin	8.90	0.11 ^d^
9	3-Phenyllactic acid	9.06	2.71 ^c^
10	Isoquercetin	9.12	0.11 ^d^
11	Cynaroside	9.12	0.05 ^d^
12	Ferulic acid	9.25	0.21 ^d^
13	Neodiosmin	9.41	0.15 ^d^
14	Formononetin	9.36	0.06 ^d^
15	Azelaic acid	9.97	1.93 ^c^
16	Glycitein	10.00	2.21 ^c^
17	Luteolin	10.83	0.16 ^d^
18	Quercetin	10.88	9.93 ^b^
19	Apigenin	11.57	14.73 ^a^
20	Naringenin	11.65	0.08 ^d^
21	Diosmetin	11.65	2.88 ^c^

Data are expressed as means ± SD (*n* = 3). Different letters represent significant difference at *p* < 0.05.

**Table 3:** Scavenging ability and inhibitory effect of phenolic compounds on α-dicarbonyl compounds.

Compounds	GO Scavenging			MGO Scavenging			3-DG Scavenging			Inhibition of Pyrraline		
	0.1 mL	0.2 mL	0.5 mL	0.1 mL	0.2 mL	0.5 mL	0.1 mL	0.2 mL	0.5 mL	0.1 mL	0.2 mL	0.5 mL
Quercetin	0.20 ± 0.03 ^b^	0.29 ± 0.02 ^b^	0.51 ± 0.04 ^a^	0.18 ± 0.02 ^c^	0.32 ± 0.01 ^b^	0.57 ± 0.07 ^a^	0.14 ± 0.01 ^b^	0.17 ± 0.03 ^b^	0.40 ± 0.05 ^a^	0.15 ± 0.02 ^b^	0.20 ± 0.02 ^b^	0.44 ± 0.05 ^a^
Neochlorogenic acid	0.17 ± 0.02 ^c^	0.23 ± 0.03 ^b^	0.47 ± 0.05 ^a^	0.14 ± 0.03 ^c^	0.29 ± 0.03 ^b^	0.50 ± 0.03 ^a^	0.13 ± 0.01 ^c^	0.25 ± 0.02 ^b^	0.41 ± 0.03 ^a^	0.11 ± 0.01 ^b^	0.19 ± 0.04 ^b^	0.41 ± 0.07 ^a^
2-Hydroxycaproic acid	0.24 ± 0.01 ^c^	0.39 ± 0.02 ^b^	0.54 ± 0.01 ^a^	0.20 ± 0.03 ^c^	0.34 ± 0.02 ^b^	0.53 ± 0.02 ^a^	0.09 ± 0.00 ^b^	0.15 ± 0.01 ^b^	0.41 ± 0.03 ^a^	0.17 ± 0.01 ^c^	0.32 ± 0.06 ^b^	0.52 ± 0.03 ^a^
Catechin	0.22 ± 0.03 ^c^	0.35 ± 0.04 ^b^	0.51 ± 0.04 ^a^	0.19 ± 0.04 ^c^	0.33 ± 0.02 ^b^	0.55 ± 0.06 ^a^	0.11 ± 0.03 ^c^	0.23 ± 0.01 ^b^	0.47 ± 0.01 ^a^	0.19 ± 0.02 ^c^	0.27 ± 0.03 ^b^	0.55 ± 0.05 ^a^
4-Hydroxyphenyllactic acid	0.21 ± 0.01 ^b^	0.30 ± 0.03 ^b^	0.50 ± 0.03 ^a^	0.15 ± 0.01 ^c^	0.32 ± 0.02 ^b^	0.51 ± 0.07 ^a^	0.18 ± 0.02 ^c^	0.27 ± 0.02 ^b^	0.44 ± 0.03 ^a^	0.16 ± 0.03 ^c^	0.24 ± 0.02 ^b^	0.43 ± 0.03 ^a^
Ferulic acid	0.22 ± 0.03 ^c^	0.33 ± 0.03 ^b^	0.53 ± 0.01 ^a^	0.19 ± 0.00 ^c^	0.37 ± 0.02 ^b^	0.52 ± 0.07 ^a^	0.09 ± 0.01 ^c^	0.18 ± 0.01 ^b^	0.37 ± 0.04 ^a^	0.15 ± 0.01 ^c^	0.26 ± 0.05 ^b^	0.51 ± 0.04 ^a^
2-Isopropylmalic acid	0.21 ± 0.02 ^c^	0.31 ± 0.02 ^b^	0.47 ± 0.04 ^a^	0.19 ± 0.01 ^c^	0.27 ± 0.02 ^b^	0.42 ± 0.07 ^a^	0.14 ± 0.03 ^c^	0.27 ± 0.05 ^b^	0.51 ± 0.02 ^a^	0.13 ± 0.01 ^c^	0.20 ± 0.02 ^b^	0.46 ± 0.05 ^a^
3-Phenyllactic acid	0.18 ± 0.01 ^c^	0.29 ± 0.04 ^b^	0.49 ± 0.03 ^a^	0.14 ± 0.02 ^c^	0.25 ± 0.02 ^b^	0.47 ± 0.02 ^a^	0.15 ± 0.03 ^c^	0.29 ± 0.03 ^b^	0.40 ± 0.02 ^a^	0.11 ± 0.01 ^b^	0.17 ± 0.08 ^b^	0.40 ± 0.04 ^a^
Rutin	0.15 ± 0.01 ^c^	0.27 ± 0.03 ^b^	0.51 ± 0.03 ^a^	0.11 ± 0.01 ^c^	0.24 ± 0.01 ^b^	0.44 ± 0.05 ^a^	0.13 ± 0.02 ^c^	0.26 ± 0.04 ^b^	0.44 ± 0.01 ^a^	0.14 ± 0.02 ^c^	0.22 ± 0.06 ^b^	0.42 ± 0.02 ^a^
Apigenin	0.18 ± 0.02 ^c^	0.27 ± 0.02 ^b^	0.48 ± 0.05 ^a^	0.10 ± 0.02 ^c^	0.18 ± 0.00 ^b^	0.51 ± 0.04 ^a^	0.16 ± 0.02 ^c^	0.29 ± 0.03 ^b^	0.43 ± 0.04 ^a^	0.14 ± 0.01 ^c^	0.27 ± 0.03 ^b^	0.41 ± 0.05 ^a^
Diosmetin	0.13 ± 0.00 ^c^	0.25 ± 0.01 ^b^	0.47 ± 0.07 ^a^	0.12 ± 0.00 ^c^	0.23 ± 0.01 ^b^	0.48 ± 0.05 ^a^	0.15 ± 0.01 ^c^	0.29 ± 0.02 ^b^	0.50 ± 0.03 ^a^	0.11 ± 0.00 ^c^	0.22 ± 0.10 ^b^	0.43 ± 0.07 ^a^
Azelaic acid	0.17 ± 0.03 ^c^	0.28 ± 0.05 ^b^	0.43 ± 0.04 ^a^	0.10 ± 0.01 ^b^	0.12 ± 0.04 ^b^	0.47 ± 0.01 ^a^	0.14 ± 0.03 ^c^	0.27 ± 0.03 ^b^	0.45 ± 0.04 ^a^	0.10 ± 0.01 ^c^	0.20 ± 0.05 ^b^	0.44 ± 0.03 ^a^
Caffeic acid	0.19 ± 0.04 ^c^	0.29 ± 0.02 ^b^	0.46 ± 0.04 ^a^	0.11 ± 0.00 ^b^	0.17 ± 0.04 ^b^	0.50 ± 0.07 ^a^	0.14 ± 0.02 ^c^	0.21 ± 0.01 ^b^	0.41 ± 0.02 ^a^	0.13 ± 0.03 ^c^	0.21 ± 0.02 ^b^	0.45 ± 0.08 ^a^

Data are expressed as means ± SD (*n* = 3). Different letters represent significant difference at *p* < 0.05.

## Data Availability

Not applicable.

## Supplementary Materials

The following are available online at https://www.mdpi.com/article/10.3390/foods10051109/s1, Figure S1: (a) UPLC chromatogram of Catechin, (a-1) MS of Catechin, (b) UPLC chromatogram of Caffeic acid, (b-1) MS of Caffeic acid, (c) UPLC chromatogram of Rutin, (c-1) MS of Rutin, (d) UPLC chromatogram of Ferulic acid, (d-1) MS of Ferulic acid, (e) UPLC chromatogram of Azelaic acid, (e-1) MS of Azelaic acid, (f) UPLC chromatogram of Apigenin, (f-1) MS of Apigenin, (g) UPLC chromatogram of Octadecenoic acid, (g-1) MS of Octadecenoic acid, (h) UPLC chromatogram of Dodecanedioic acid, (h-1) MS of Dodecanedioic acid (i) UPLC chromatogram of Ibufenac, (i-1) MS of Ibufenac, (j) UPLC chromatogram of Dihome, (j-1) MS of Dihome., Figure S2: (a) UPLC chromatogram of Caffeic acid, (b-1) MS of Caffeic acid, (c) UPLC chromatogram of 2-Hydroxycaproic acid, (c-1) MS of 2-Hydroxycaproic acid, (d) UPLC chromatogram of Astilibin, (d-1) MS of Astilibin, (g) UPLC chromatogram of Rutin, (g-1) MS of Rutin, (e) UPLC chromatogram of 3-Phenyllactic acid, (e-1) MS of 3-Phenyllactic acid, (f) UPLC chromatogram of Isoquercetin, (f-1) MS of Isoquercetin, (g) UPLC chromatogram of Cynaroside, (g-1) MS of Cynaroside, (h) UPLC chromatogram of Ferulic acid, (h-1) MS of Ferulic acid, (i) UPLC chromatogram of Neodiosmin, (i-1) MS of Neodiosmin, (j) UPLC chromatogram of Formononetin, (j-1) MS of Formononetin, (k) UPLC chromatogram of Azelaic acid, (k-1) MS of Azelaic acid, (l) UPLC chromatogram of Glycitein, (l-1) MS of Glycitein, (m) UPLC chromatogram of Luteolin, (m-1) MS of Luteolin, (n) UPLC chromatogram of Quercetin, (n-1) MS of Quercetin, (o) UPLC chromatogram of Apigenin, (o-1) MS of Apigenin, (p) UPLC chromatogram of Naringenin, (p-1) MS of Naringenin, (q) UPLC chromatogram of Diosmetin, (q-1) MS of Diosmetin.
