# Supplementary material for: Highland Barley and Its By-Products Enriched with Phenolic Compounds for Inhibition of Pyrraline Formation by Scavenging α-Dicarbonyl Compounds
Source: Foods. 2021 May 17;10(5):1109. doi: 10.3390/foods10051109 (PMC8156036; doi:10.3390/foods10051109)
Supplement: Supplementary file 1 [file foods-10-01109-s001.zip › foods-1195888-supplementary.pdf]

Supplementary Materials

Highland Barley and Its By-Products Enriched with Phenolic Compounds for Inhibition of Pyrraline Formation by Scavenging α-Dicarbonyl Compounds

Dianwei Zhang, Pei Zhu, Luxuan Han, Xiaomo Chen, Huilin Liu * and Baoguo Sun

Beijing Advanced Innovation Center for Food Nutrition and Human Health, Beijing Engineering and Technology Research Center of Food Additives, Beijing Technology and Business University, Beijing 100048, China

* Correspondence: liuhuilin@btbu.edu.cn; Tel.: +86-10-68984545

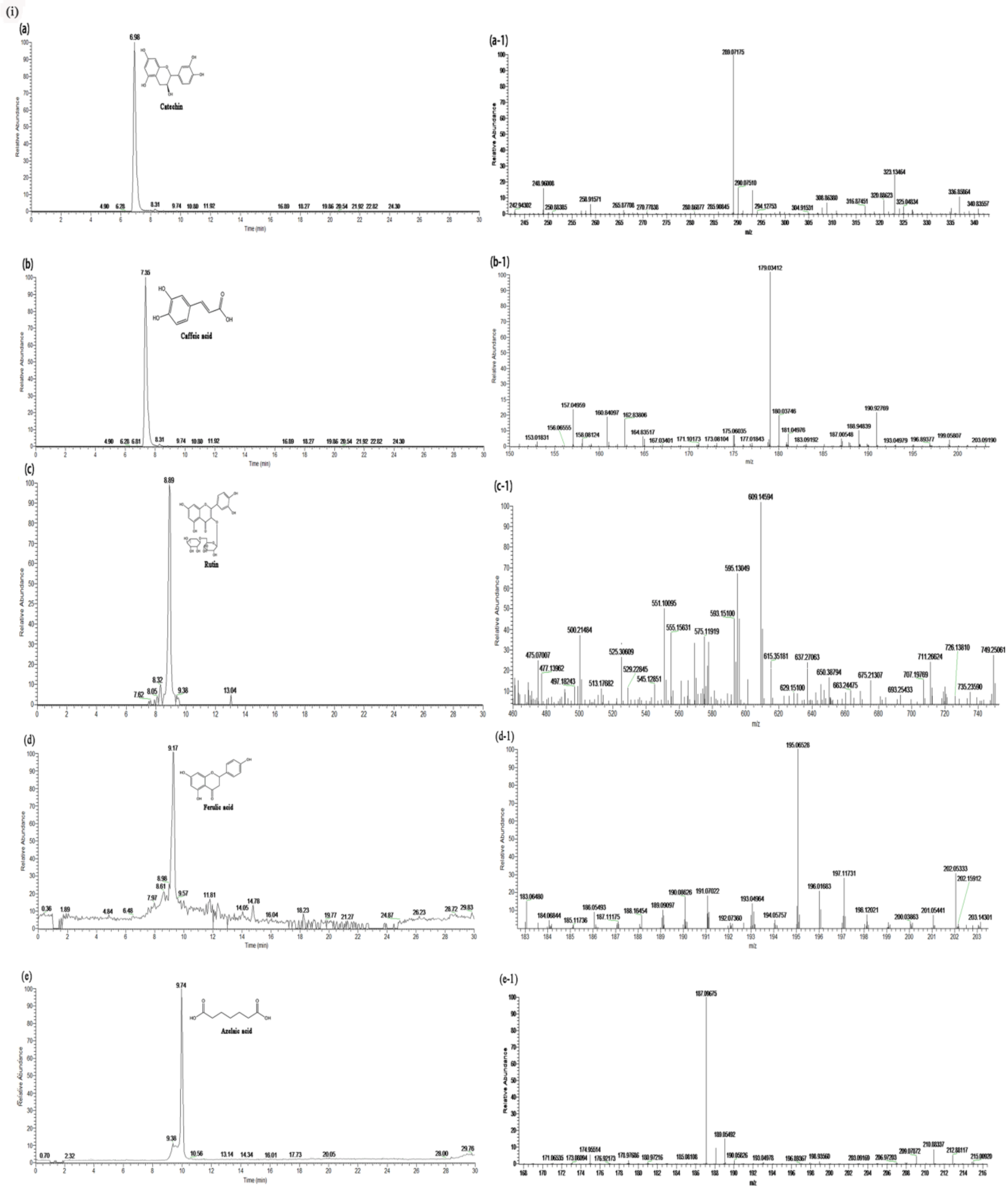
(i)
(a)
Catechin
(a-1)
(b)
Caffeic acid
(b-1)
(c)
Rutin
(c-1)
(d)
Ferulic acid
(d-1)
(e)
Azelaic acid
(e-1)
Relative Abundance
Time (min)
m/z

(ii)

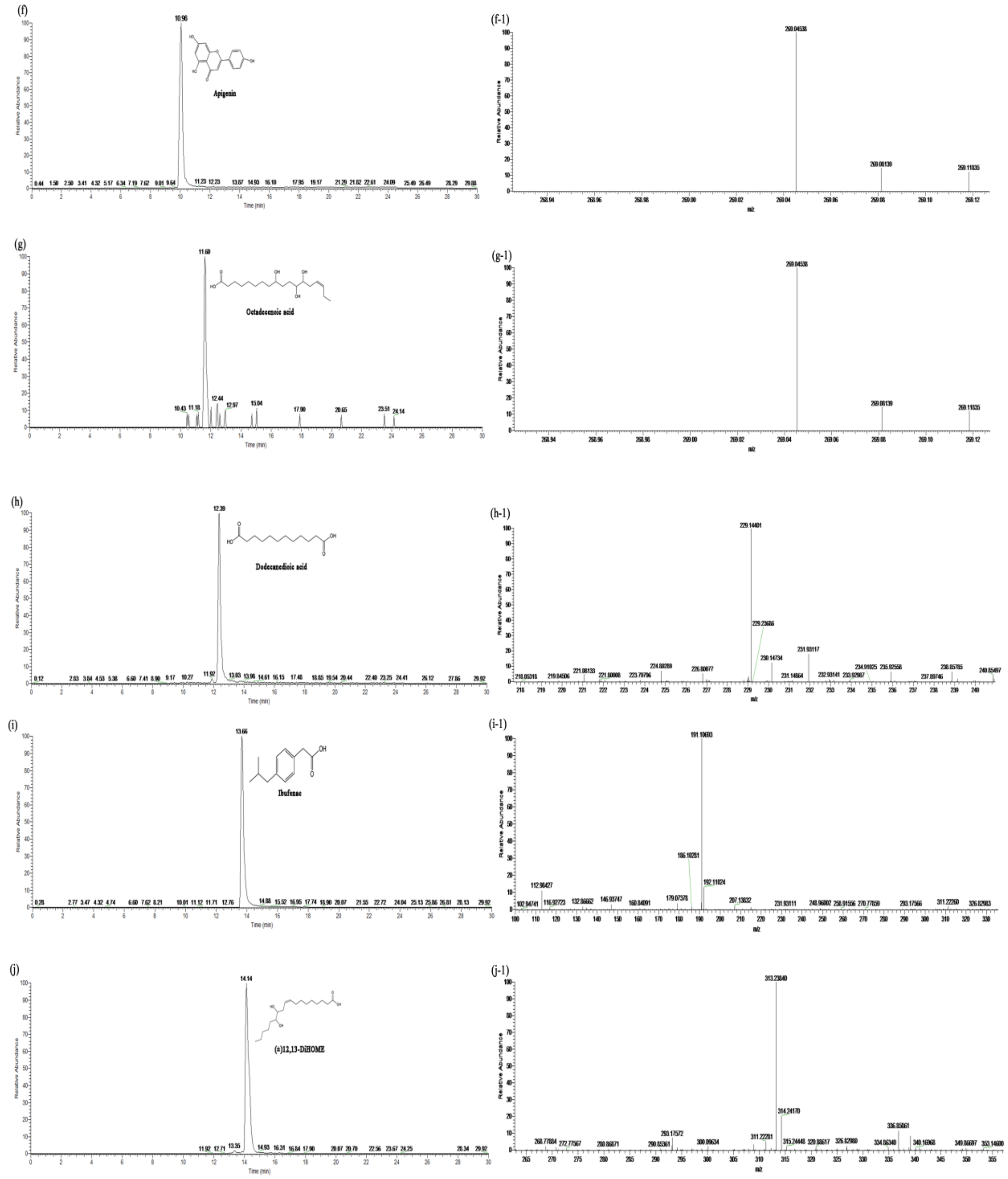

Figure S1. (a) UPLC chromatogram of Catechin, (a-1) MS of Catechin, (b) UPLC chromatogram of Caffeic acid, (b-1) MS of Caffeic acid, (c) UPLC chromatogram of Rutin, (c-1) MS of Rutin, (d) UPLC chromatogram of Ferulic acid, (d-1) MS of Ferulic acid, (e) UPLC chromatogram of Azelaic acid, (e-1) MS of Azelaic acid, (f) UPLC chromatogram of Apigenin, (f-1) MS of Apigenin, (g) UPLC chromatogram of Octadecenoic acid, (g-1) MS of Octadecenoic acid, (h) UPLC chromatogram of Dodecanedioic acid, (h-1) MS of Dodecanedioic acid (i) UPLC chromatogram of Ibufenac, (i-1) MS of Ibufenac, (j) UPLC chromatogram of Dihome, (j-1) MS of Dihome.

(i)

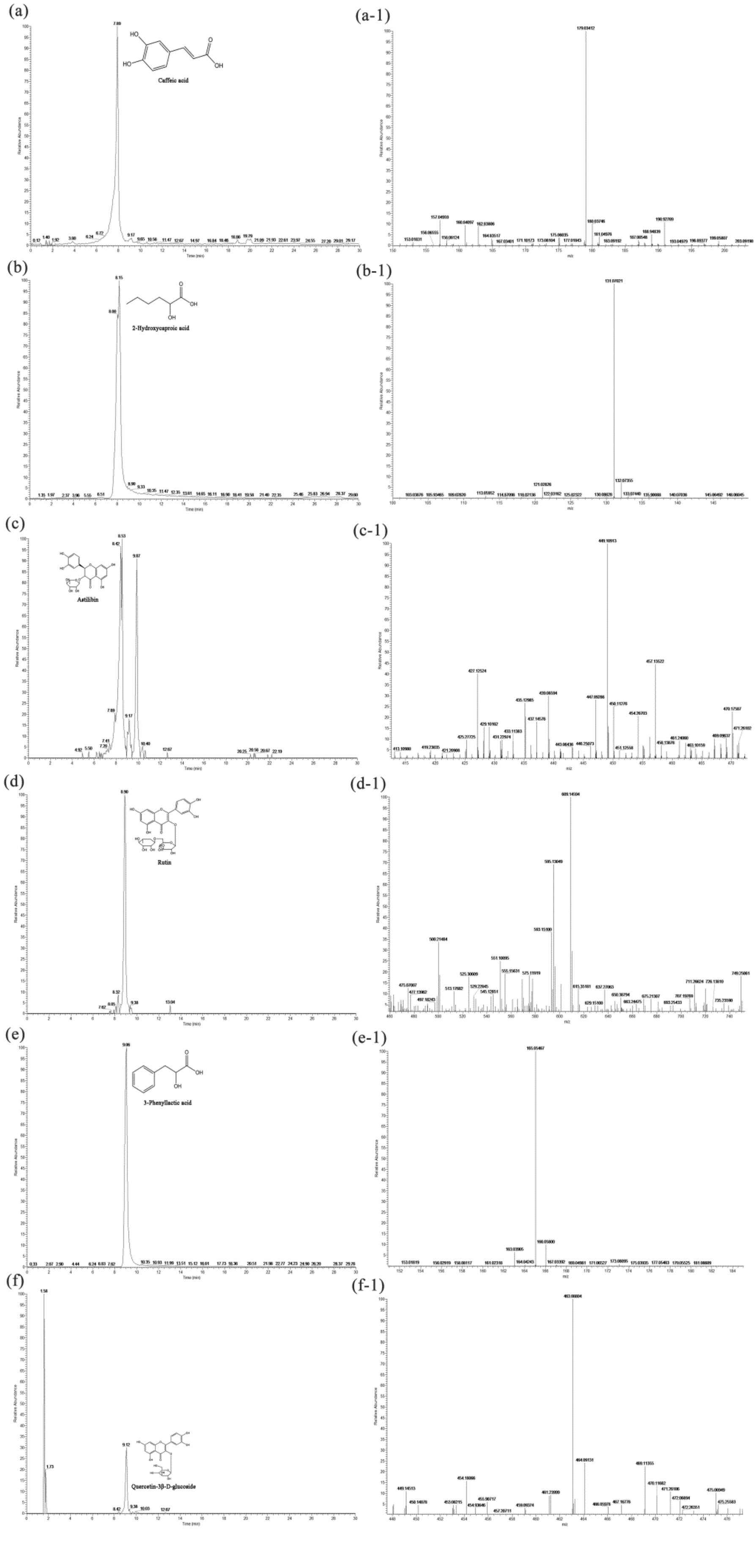
(a)
(a-1)
Caffeic acid
(b)
(b-1)
2-Hydroxycaproic acid
(c)
(c-1)
Astilibin
(d)
(d-1)
Rutin
(e)
(e-1)
3-Phenyllactic acid
(f)
(f-1)
Quercetin-3β-D-glucoside

(ii)

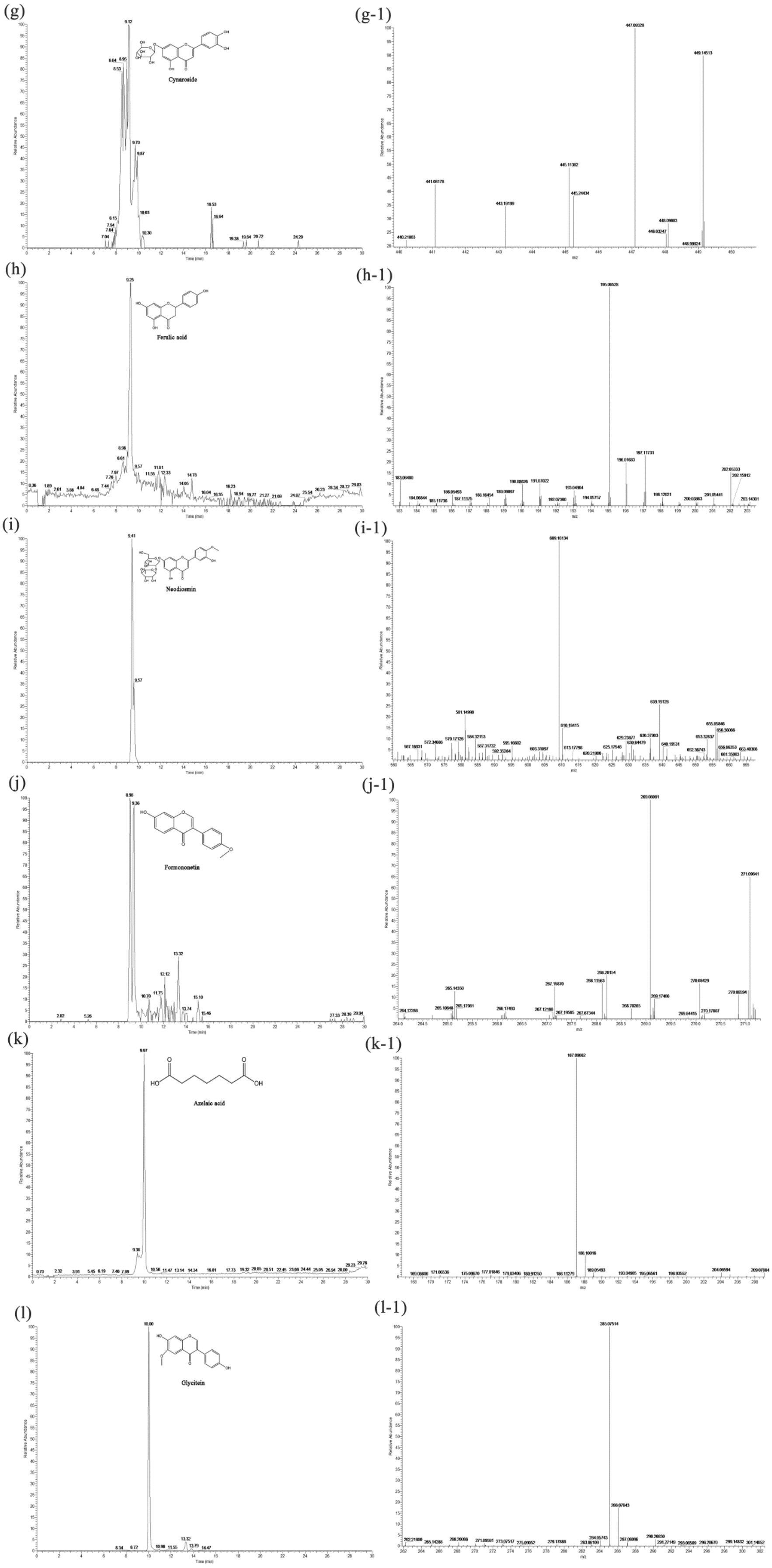
(g)
Cynaroside
(g-1)
(h)
Ferulic acid
(h-1)
(i)
Neodiosmin
(i-1)
(j)
Formononetin
(j-1)
(k)
Azelaic acid
(k-1)
(l)
Glycitein
(l-1)

(iii)

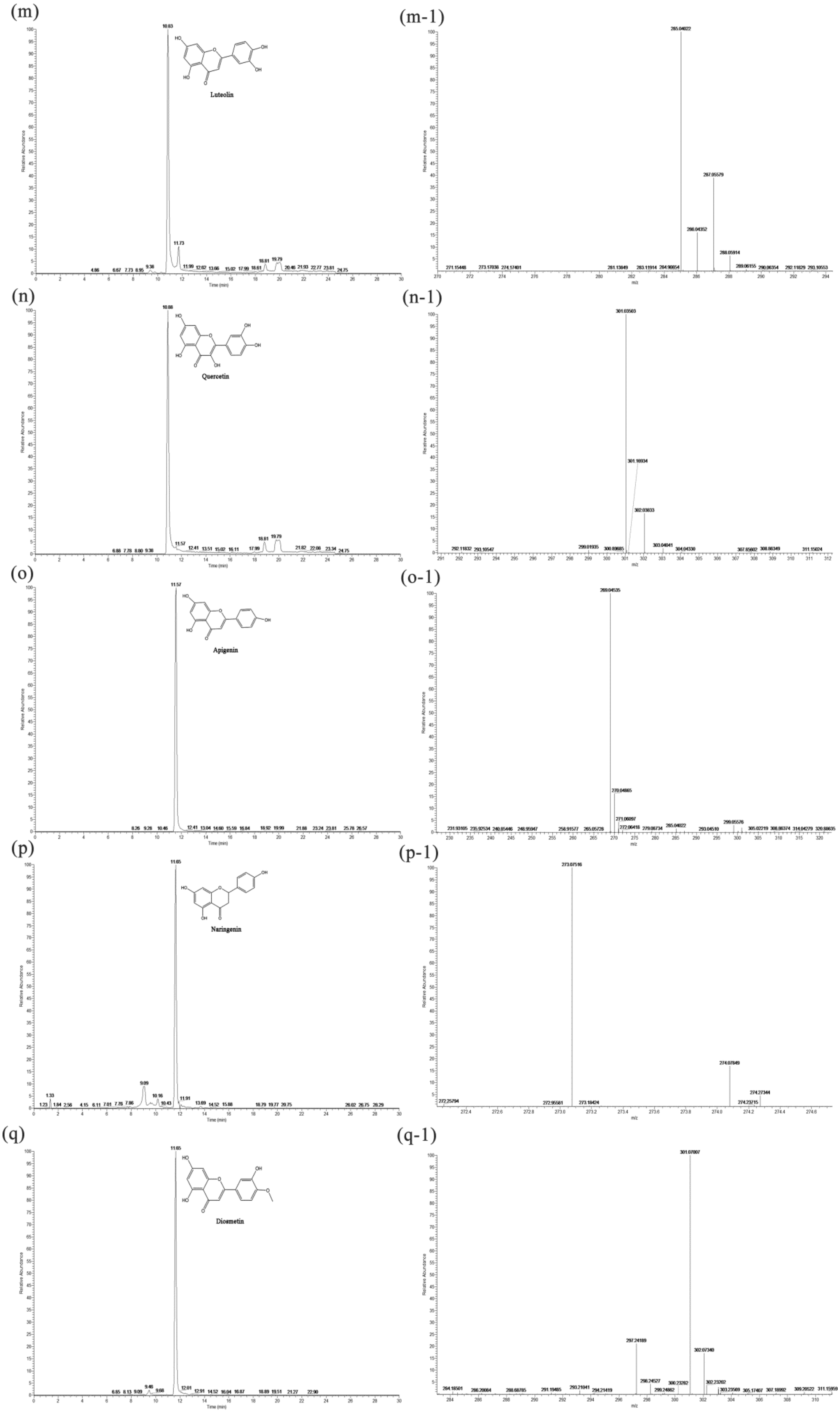
(m)
Luteolin
(m-1)
285.04022
287.05579
(n)
Quercetin
(n-1)
301.03503
(o)
Apigenin
(o-1)
269.04535
(p)
Naringenin
(p-1)
273.07516
(q)
Diosmetin
(q-1)
301.07007
Relative Abundance
Time (min)
m/z

Figure S2. (a) UPLC chromatogram of Caffeic acid, (b-1) MS of Caffeic acid, (c) UPLC chromatogram of 2-Hydroxycaproic acid, (c-1) MS of 2-Hydroxycaproic acid, (d) UPLC chromatogram of Astilibin, (d-1) MS of Astilibin, (g) UPLC chromatogram of Rutin, (g-1) MS of Rutin, (e) UPLC chromatogram of 3-Phenyllactic acid, (e-1) MS of 3-Phenyllactic acid, (f) UPLC chromatogram of Isoquercetin, (f-1) MS of Isoquercetin, (g) UPLC chromatogram of Cynaroside, (g-1) MS of Cynaroside, (h) UPLC chromatogram of Ferulic acid, (h-1) MS of Ferulic acid, (i) UPLC chromatogram of Neodiosmin, (i-1) MS of Neodiosmin, (j) UPLC chromatogram of Formononetin, (j-1) MS of Formononetin, (k) UPLC chromatogram of Azelaic acid, (k-1) MS of Azelaic acid, (l) UPLC chromatogram of Glycitein, (l-1) MS of Glycitein, (m) UPLC chromatogram of Luteolin, (m-1) MS of Luteolin, (n) UPLC chromatogram of Quercetin, (n-1) MS of Quercetin, (o) UPLC chromatogram of Apigenin, (o-1) MS of Apigenin, (p) UPLC chromatogram of Naringenin, (p-1) MS of Naringenin, (q) UPLC chromatogram of Diosmetin, (q-1) MS of Diosmetin.
